# Clear cell chondrosarcoma of the head and neck

**DOI:** 10.1186/1758-3284-4-13

**Published:** 2012-04-20

**Authors:** Sepideh Mokhtari, Abbas Mirafsharieh

**Affiliations:** 1 Department of Oral and Maxillofacial Pathology, Shahid Beheshti University of Medical Sciences, Velenjak Street, Tehran, Iran; 2 Department of Pathology, Shahid Beheshti University of Medical Sciences, Tehran, Iran

**Keywords:** Clear cell chondrosarcoma, head and neck, histopathology

## Abstract

Clear cell chondrosarcoma is a rare variant of chondrosarcoma that mostly involves the end of long bones. However, nine cases have been reported in the head and neck: four in larynx, two in nasal septum, two in maxilla and one in the skull. These cases form the basis of this review. Head and neck cases accounts for less than 5% of Clear cell chondrosarcomas in the whole body and the larynx is the most common place. The histological findings of head and neck cases are consistent with general features of this entity in the whole body and nearly all tumors in this case series had a component of conventional chondrosarcoma. Clear cell chondrosarcoma is an intracompartmental tumor and retains "Grenz zone" just beneath the epithelium. Therefore, the overlying mucosa remained intact in all laryngeal cases. Nasal tumor caused ballooning of the septum and the maxillary lesion did not involve the oral mucosa. This tumor presents various radiographic features in the head and neck area. Chondroblastoma, chondroma, osteoblastoma, osteosarcoma and metastatic renal cell carcinoma are included in the histologic differential diagnoses. Differentiation from chondroblastic osteosarcoma is important in the maxilla. A wide resection is adequate in most cases. However, some laryngeal cases show tendency to recur. Clear cell chondrosarcoma is a slow growing tumor and this necessitates a long time follow-up of patients. Due to the extreme rarity in the head and neck, diagnosis of Clear cell chondrosarcoma in this area, must be confirmed by histochemical and immunohistochemical studies.

## Introduction

Approximately 1% to 3% of all chondrosarcomas occur in the head and neck area and they arise frequently in the maxilla, mandible, skull, cervical vertebrae, nasal cavity and larynx [1]. The clear cell chondrosarcoma is an extremely rare variant of chondrosarcoma that accounts for about 2% of all chondrosarcomas. This mesenchymal tumor usually affects the epiphyseal region or apophysis of the long bones particularly the femur and humerus. Patients are more in the third, fourth or fifth decade of life [2]. Local pain is the predominant symptom and the duration of pain is more than 1.5 years in many cases. This rare cartilaginous tumor is a low-grade malignancy that does not invade soft tissues in many cases. However, "en bloc" resection is the preferred choice of treatment in all cases. Lesions may recur even after two decades of first surgical treatment. Lung, brain, and skeleton are the most probable metastatic locations [3]. A summary of clinical data for this rare entity is provided in Table 1.

**Table 1:** A summary of clinical data for clear cell chondrosarcoma

Clear Cell Chondrosarcoma	
**Clinical Features**	An extremely rare variant of chondrosarcoma
	
	More in adults between 3-5 decades of life
	
	Male predominance
	
	Usually in the proximal femur and humerus
	
	Long-standing symptoms as pain, pathologic fracture,...

**Radiographic appearance**	Not diagnostic, may mimic giant cell tumor or chondroblastoma

**Behavior**	Low-grade behavior
	
	Clear tendency for late recurrence and metastases
	
	Few cases behave aggressively

**Treatment**	"en bloc" resection
	
	A long time follow-up is necessary

To the best of our knowledge, only nine cases of this rare entity in the head and neck area have been reported. These cases form the basis of this review.

## Materials and methods

We reviewed MEDLINE-indexed publications using the keyword "clear cell chondrosarcoma". We found more than 200 reported cases of clear cell chondrosarcoma in the literature. Among these cases, nine cases had occurred in the head and neck area: Four in larynx, two in nasal septum, two in maxilla and one in skull. Available clinical and histological data of these cases were extracted from the relevant articles [4-12] and summarized in Table 2. No clinical or histological data was available in the articles [11,12] for two cases of clear cell chondrosarcoma in the head and neck (case 8, 9).

**Table 2:** Clinical Data of Patients with Head and Neck Clear Cell Chondrosarcoma

Case	Age/ sex	Site	Symptoms	Durationof symptoms	Treatment	Recurrence	Further treatment	Follow up
1	50/F	Maxilla	Swelling & slow enlargement	3 y	Local excision	No	-	10 y

2	79/F	Nasal septum	Nasal obstruction	6 m	Resection	No	-	16 m

3	62/F	Nasal septum	Recurrent epistaxis	1 m	Wide resection	No	-	30 m

4	46/M	Subglottic	Dyspnea & Stridor	6 m	Laryngectomy	No	-	6 m

5	61/M	Subepiglott	Dyspnea & Hoarseness	History of chondroma 22 yr before	Laryngectomy& Neck dissection& Radiotherapy	No	-	N/A

6	57/M	Cricoid cartilage	Dyspnea	-	Laryngectomy & Neck dissection& Hemithyroidectomy	1-15 m 2-20 m 3-33 m	Locally removed	3 y

7	56/M	Thyroid cartilage	1-Voice change 2-Change in neck shape	1-2 yr 2- several mo	Partial resection of thyroid cartilage	-	-	4 y

8	N/A	Maxilla	N/A	N/A	N/A	N/A	N/A	N/A

9	N/A	Skull	N/A	N/A	N/A	N/A	N/A	N/A

## Results

### Clinical features

Three patients were men and three were women. The median age of patients was approximately 59 years (range 46-79 years). The duration of symptoms was variable, in a range of 1 month to 3 years. One of laryngeal cases (case 5) had a history of chondroma for 22 years before true diagnosis. This lesion recurred three times until the diagnosis of clear cell chondrosarcoma was made [8]. Patients with laryngeal tumors had symptoms as dyspnea, stridor, hoarseness and change in the neck shape. The overlying mucosa was intact in all laryngeal cases. Patients with nasal involvement complained of nasal obstruction or recurrent epistaxis. There was a huge ballooning of nasal septum in one case [5]. The Maxillary lesion presented with a painless enlarging lesion in the anterior area and the overlying buccal mucosa was not attached to the tumor [4]. Lymph node involvement was clinically evident in some cases.

### Radiographic appearance

Tumors presented primarily as lobulated, multilocular or even well-defined cyst like radiolucencies. In laryngeal and nasal tumors, areas of intratumoral calcification were seen. The maxillary lesion was a poorly defined, osteolytic lesion between the roots of teeth and no root resorption was seen. Localized thickening of the periodontal ligament space is a typical feature of jaw chondrosarcoma, but it was not present in this maxillary case [4].

### Gross appearance

The tumor was 10 cm in the greatest diameter [8]. Tumors frequently revealed a prominent firm lobular pattern of growth. They had friable translucent cartilaginous nodules mixed with soft, gray or blue cut surface similar to gross appearance of conventional chondrosarcoma.

### Histological features

**Figure 1:**
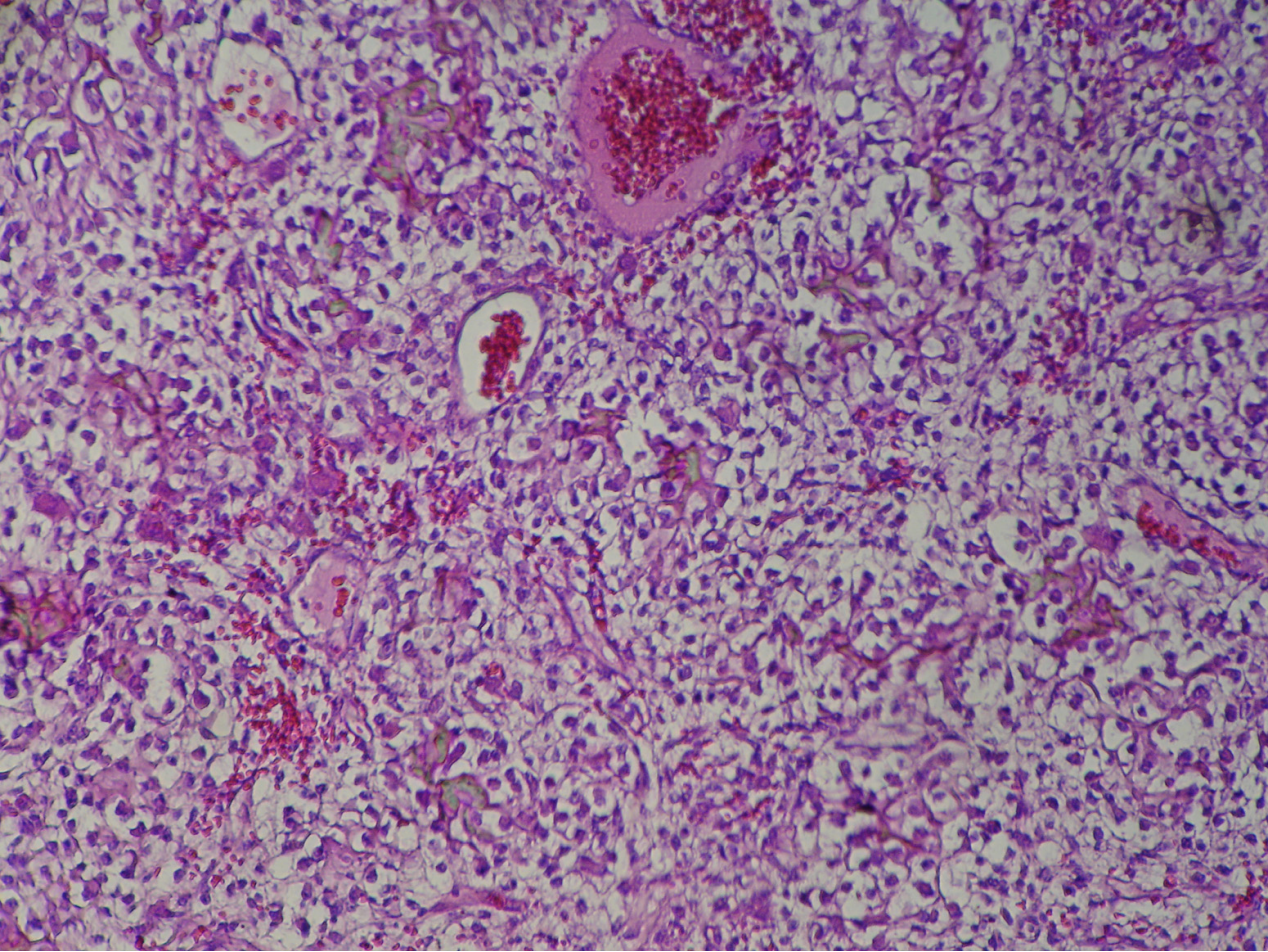
**Sheet-like arrangement of tumor cells in clear cell chondrosarcoma**. Tumor cells have clear cytoplasm, hyperchromatic nucleus and distinct cytoplasmic borders.

**Figure 2:**
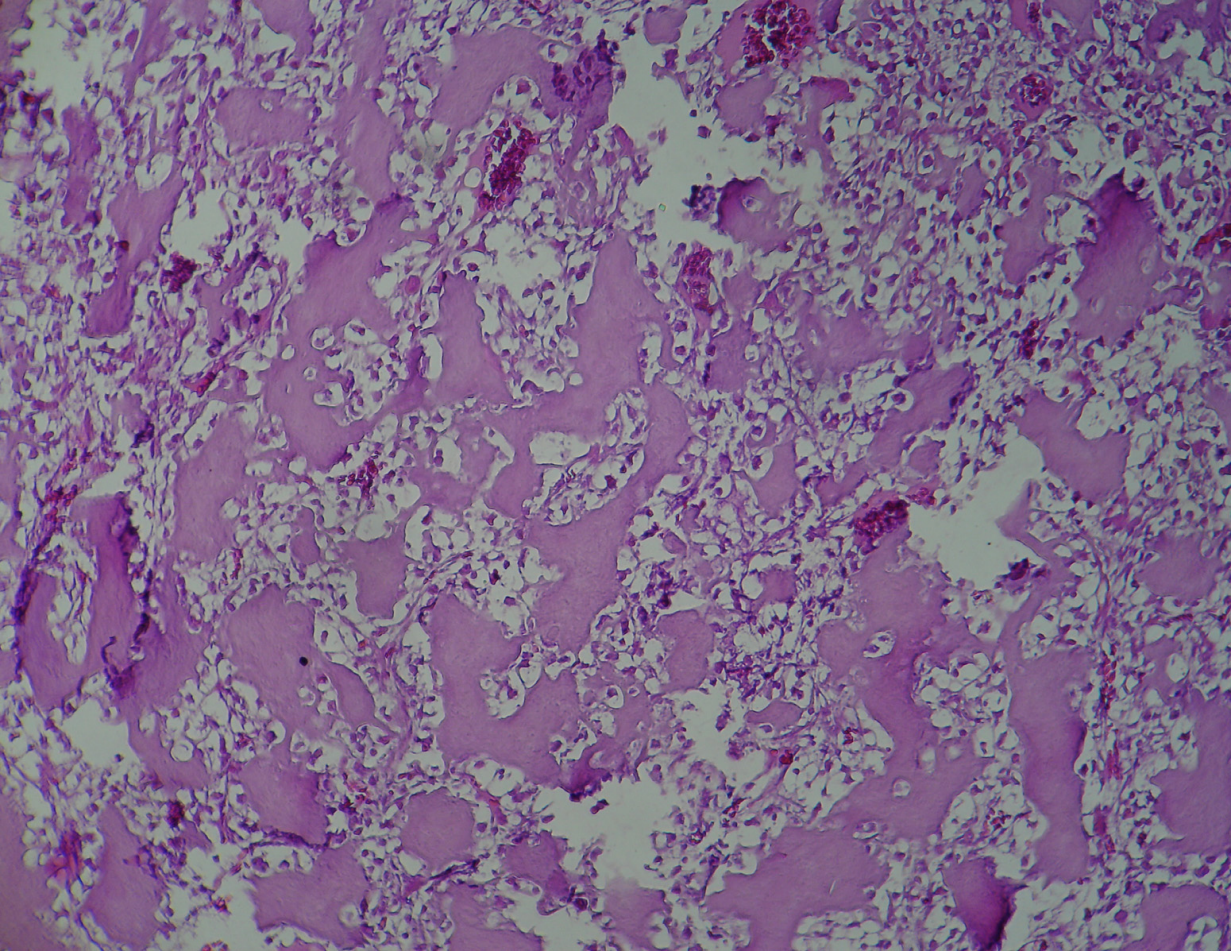
**Ossification within clear cell chondrosarcoma**.

The histological findings were consistent with general features of clear cell chondrosarcoma (Figures 1,2) characterized by a lobulated (in most cases) neoplasm. Tumors were made up of uniform to polymorphic densely-packed large cells, side by side or merging with each other. The cells had an abundant round to polygonal clear cytoplasm with small hyperchromatic to large round central nucleus, lacking production of hyaline chondroid matrix. Various cellular differentiations of tumor cells were also seen. Frequently, a group of clear cells had a faintly to intensively acidophilic granular cytoplasm resembling chondroblasts. Nearly all tumors had a component of conventional chondrosarcoma. Characteristically, the cartilage tumor cells in these fields had produced large amounts of mature chondroid matrix with multiple foci of calcification. However, chondroid matrix formation was rare within the clear cell component. Multinucleated osteoclast-like giant cells were present in most cases, but not within the compact fields of clear cells. The presence of theses giant cells is also absent in some recently reported cases of clear cell chondrosarcoma and it is not necessary for diagnosis [13]. The mitotic rate was very low within all cases. Frequently, irregularly shaped trabeculae of osteoid and woven bone were scattered throughout the lesions. The tumor cells showed well-defined pushing borders or infiltration beyond the organ borders to the surrounding soft or hard tissues. Small amounts of intracellular glycogen were demonstrated by positive PAS staining in some cases whereas some others were negative.

### Treatment

Nasal tumors were resected and the maxillary lesion was locally excised. Laryngectomy was performed in 75% (3 of 4) of laryngeal cases and additional radical neck dissection was done in 50% (2 of 4). Locally removal of the tumor as a chondroma before its true diagnosis in case5 led to two recurrences. Radiotherapy was done in only one case and the average follow up of patients was 42 month (range from 6 months to 10 years).

## Discussion

Less than 5% (9/200) of reported cases of clear cell chondrosarcoma have occurred in the head and neck area, and although, laryngeal chondrosarcoma is a rare entity, the larynx is the most common place involved by clear cell type in the head and neck (44%). The mean age of reported cases of clear cell chondrosarcoma in the head and neck is similar to the mean age of chondrosarcoma in the whole body (60 years). However this differs from the average age of patients with head and neck chondrosarcoma that is mostly 35 to 45 years old [2].

Although a definite male predominance is seen in various case series of clear cell chondrosarcoma; this propensity is not observed in this case series of head and neck. Head and neck clear cell chondrosarcomas have occurred in alveolar bone, nasal septum and larynx where are among the common sites of head and neck chondrosarcoma.

Many clear cell chondrosarcomas are intracompartmental rather than extracompartmental; so they expand the bones without permeating the cortex [14]. They also, similar to other primary mesenchymal neoplasms, retain "Grenz zone" of tumor-free superficial submucosa just beneath the epithelium. Therefore, nasal tumor in case 2 caused ballooning of the septum; maxillary lesion did not involve the oral mucosa; and despite the lumen obstruction by tumor, the overlying mucosa remained intact in all laryngeal cases.

The radiographic appearance of clear-cell chondrosarcoma is generally an osteolytic destructive expansive lesion with sharp margins; however, in larger tumors, the margins become poorly defined [4]. Punctuate densities are also occasionally identified within the tumor.

Tumors of this case series in head and neck presented various radiographic features from unilocular to multilocular and from well-differentiated to ill-defined lesions. Intratumoral calcification that was present in some cases is mostly related to the presence of newly formed osseous elements. Clear cell chondrosarcoma is frequently diagnosed as chondroblastoma radiographically and histologically in literature and could be its malignant counterpart [15]. Since chondroblastoma has not been reported in the maxilla or nasal skeleton [4] the transformation of chondroblastoma to clear cell chondrosarcoma in cases of maxillofacial skeleton seems improbable.

Because of osteoid formation, bone-forming lesions such as osteoblastoma or osteosarcoma are included in the histologic differential diagnoses [14]. This is mainly important in anatomic sites of head and neck that are more prone to osteosarcoma such as skull, maxilla and mandible. Chondroblastic osteosarcoma is the most common variant of this tumor in the maxilla and therefore, it is one of main differential diagnoses of clear cell chondrosarcoma of maxilla. Osteosarcoma must be differentiated by the absence of clear cells, the presence of severe nuclear polymorphism and atypical mitoses. Three cases of high grade osteosarcoma with significant areas of clear cells have been reported [16]. Although extremely rare, but this entity must also be considered. The other differential diagnoses, especially in laryngeal neoplasms, include squamous cell carcinoma and metastatic clear cell carcinoma mainly renal cell carcinoma [9].

Areas of conventional chondrosarcoma are often present in cases of clear cell chondrosarcoma and this component was present in almost all of these cases. Recent findings indicate that chondroma and chondrosarcoma of the larynx are closely related and it is well known that distinction between these two entities could be extremely difficult [17,18]; similar to the case 5, it is not uncommon for the biopsy tissue diagnosed as a benign chondroma to be ultimately diagnosed as chondrosarcoma[19]. Since the occurrence of a true chondroma of the larynx is extremely rare [2], and because more than 90% of all laryngeal cartilaginous tumors are chondrosarcomas, cartilage lesions that are clinically significant or have recurred should be considered as chondrosarcoma [20,21]. As well, benign chondrogenic tumors of the jaws are quite rare and benign appearing cartilaginous tumors in the jaws are considered potentially malignant.

Wide and aggressive surgical resection is the critical point in treatment of head and neck chondrosarcoma and results in a favorable prognosis. Metastases are not common in head and neck chondrosarcoma [2]. The 5-year survival rate for chondrosarcoma of the head and neck varies from 43% to 95% [2]. Clear cell variant is a distinct low-grade sarcoma with potential for local recurrence or distant metastasis. Recurrence rate of 19% has been described in some case series of this tumor [22]. It has been stated that regardless of the stage of tumor, a wide margin of normal bone and soft tissue around the resected lesion is required to achieve adequate local control [14]. However, some patients in this series, whose lesions were treated with local removal, did not developed local recurrences or metastases. Although proton beam therapy has been successfully used in the treatment of head and neck chondrosarcoma [23], its effectiveness in clear cell variant of head and neck chondrosarcoma needs more investigation.

The prognosis was excellent in nasal and maxillary lesions. The absence of root resorption was also consistent with indolent clinical course of the maxillary tumor. Recurrence was noted in two laryngeal cases and it seems that laryngeal tumors have more probability to recur. Therefore, they need more consideration. Although, total laryngectomy is reserved for high-grade laryngeal chondrosarcomas, some studies indicate that conservative therapeutic approach in conventional chondrosarcoma has increased the incidence of local recurrence from 77% to 86% [24]. Two of four presented laryngeal cases were treated with laryngectomy and radical neck dissection and this was consistent with their clinical course. Nevertheless, the behavior of all four laryngeal tumors supports the general experience that chondrosarcomas in the larynx have a favorable course of disease [24,25]; as well, early correct diagnosis is important to avoid multiple recurrences[9].

The primary symptoms in clear cell chondrosarcoma are generally of long duration. 55% of the patients in the largest series of clear cell chondrosarcoma have symptoms for more than 1 year [12]. This long duration of symptoms before diagnosis is consistent with a slow-growing tumor [14]. One laryngeal tumor (case 5) has an indolent clinical course over 22 years with multiple recurrences [5]. This behavior necessitates a long time follow-up of patients to judge the efficacy of treatment [26].

On the molecular level, recent studies have shown the presence of extra copies of chromosome 20 and loss or rearrangements of 9p in clear cell chondrosarcoma. In addition, expression of PTHLH, PDGFIHH, Runt-related transcription factor2 [27] have been found. Widespread positivity for matrix metalloproteinase 2 (MMP2) have been shown in some reported cases of clear cell chondrosarcoma with aggressive clinical behavior [28]. Park et al. have investigated the role of p53 in the pathogenesis of this tumor. They showed that a genetic alteration of p53 is a rare event, whereas its overexpression may occur in a substantial percentage of clear cell chondrosarcoma [29]. Allelic loss at chromosome 18q21 has been found in the laryngeal case of clear cell chondrosarcoma. This has not been described for chondrosarcomas so far [9]. For that reason, more investigations in future are necessary to determine the genetics of head and neck clear cell chondrosarcoma.

Because of the rarity in head and neck area, diagnosis of clear cell chondrosarcoma has to be confirmed by light microscopic appearance, presence of glycogen (in most cases), expression of S-100 protein and collagen type II, which is as a characteristic of clear cell chondrosarcoma, by immunohistochemistry [27]. This allows a reliable diagnosis of the rare cases of this tumor in the unusual location of head and neck; so, possible overtreatment or undertreatment of patients will be prevented.

## Conclusions

Head and neck clear cell chondrosarcoma accounts for less than 5% of clear cell chondrosarcoma in the whole body. Nine cases have been reported in larynx, nasal septum, maxilla and skull. Larynx is the most common place involved by this entity in the head and neck. The mean age of clear cell chondrosarcoma in the head and neck is similar to that of chondrosarcoma in the whole body. These tumors present with various radiographic features in the head and neck. Areas of conventional chondrosarcoma were present in almost all head and neck cases. Histological differential diagnose with other bone forming lesions such as osteoblastoma or osteosarcoma must be considered in the head and neck. A wide resection is adequate in most cases. However, some laryngeal cases show tendency to recur. Due to the extreme rarity of this tumor in the head and neck, its diagnosis must be confirmed by histochemical and immunohistochemical studies. Therefore, possible overtreatment or undertreatment of patients will be prevented.

## Competing interests

The authors declare that they have no competing interests.

## Authors' contributions

SM prepared the manuscript. AM provided advice and guidance on the pathological aspects of the paper. Both authors read and approved the final manuscript.
